# Subcostal bilateral internal thoracic artery harvesting with the da Vinci Single Port system: an experimental feasibility study

**DOI:** 10.1093/icvts/ivae071

**Published:** 2024-04-16

**Authors:** Hubert Stein, Volkmar Falk, Joseph Zacharias, Michael Ikeda, John Michael Smith, Georgia Crichton, Louis Ryckembusch, Jörg Kempfert

**Affiliations:** Intuitive Surgical Inc., Sunnyvale, CA, USA; Department of Cardiothoracic and Vascular Surgery, Deutsches Herzzentrum der Charité, Berlin, Germany; German Center of Cardiovascular Research, Partner Site, Berlin, Germany; Department of Health Science and Technology, ETH Zurich, Zurich, Switzerland; Department of Cardiothoracic Surgery, Blackpool Victoria Hospital, Blackpool, UK; Intuitive Surgical Inc., Sunnyvale, CA, USA; Department of Heart and Vascular Surgery, The Christ Hospital, Cincinnati, OH, USA; Intuitive Surgical Inc., Sunnyvale, CA, USA; Intuitive Surgical Inc., Sunnyvale, CA, USA; Department of Cardiothoracic and Vascular Surgery, Deutsches Herzzentrum der Charité, Berlin, Germany

**Keywords:** Single Port, Internal mammary artery, Bilateral, Subcostal, Internal thoracic artery, Robotic, da Vinci

## Abstract

We evaluated the feasibility of harvesting bilateral internal thoracic arteries with the da Vinci Single Port system (SP) through a single left-sided subcostal incision. Complete bilateral mobilization with sufficiently long conduits for multivessel grafting was possible in 2 human cadavers and 2 live porcine. Creating the subcostal access and docking the SP system took between 14 and 21 min and the total harvest time ranged from 65 to 125 min in all models. No major bleeding was observed in the live porcine and hemostasis was managed with the available instrumentation. One porcine deceased during surgery due to ventricular fibrillation followed by cardiac arrest. The robotic harvesting was technically easily reproduced by the surgeons and required no additional rib-spreading. Further studies will be required to assess if this subcostal approach with the da Vinci SP system yields true clinical benefits in patients.

## Graphical abstract

**Figure:**
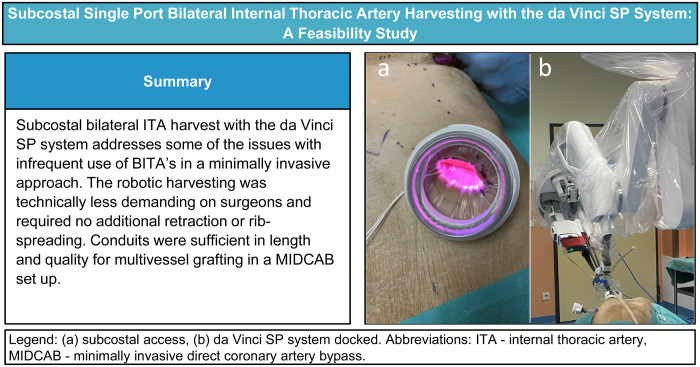

## INTRODUCTION

Using multiple arterial grafts during coronary artery bypass grafting is associated with low mortality, morbidity, and better long-term outcomes [1, 2] when compared to single arterial grafting. While use of the left internal thoracic artery (ITA) is routine worldwide, applying bilateral ITA (BITA) for full arterial revascularization is limited although recommended in current guidelines [3, 4]. BITA harvesting is considered technically more demanding [5], time-consuming, and associated with an increased rate of sternal wound infections [1, 2]. Lateral thoracotomy approaches require rib spreading of at least 25–40 mm for sufficient exposure to harvest both ITAs [6]. Hence, minimally invasive options for patients needing arterial grafting that reduce trauma, complications, and pain are scarce.

Initial studies for thoracic surgery through subxiphoid and subcostal approaches showed promising results with the da Vinci Single Port (SP) surgical system (Intuitive Inc., Sunnyvale, USA) [7, 8]. Based on these experiences a left subcostal approach was explored to evaluate the feasibility of mobilizing BITA and assess if conduit length was sufficient for multivessel minimally invasive direct coronary artery bypass (MIDCAB).

## MATERIALS AND METHODS

**Figure 1:**
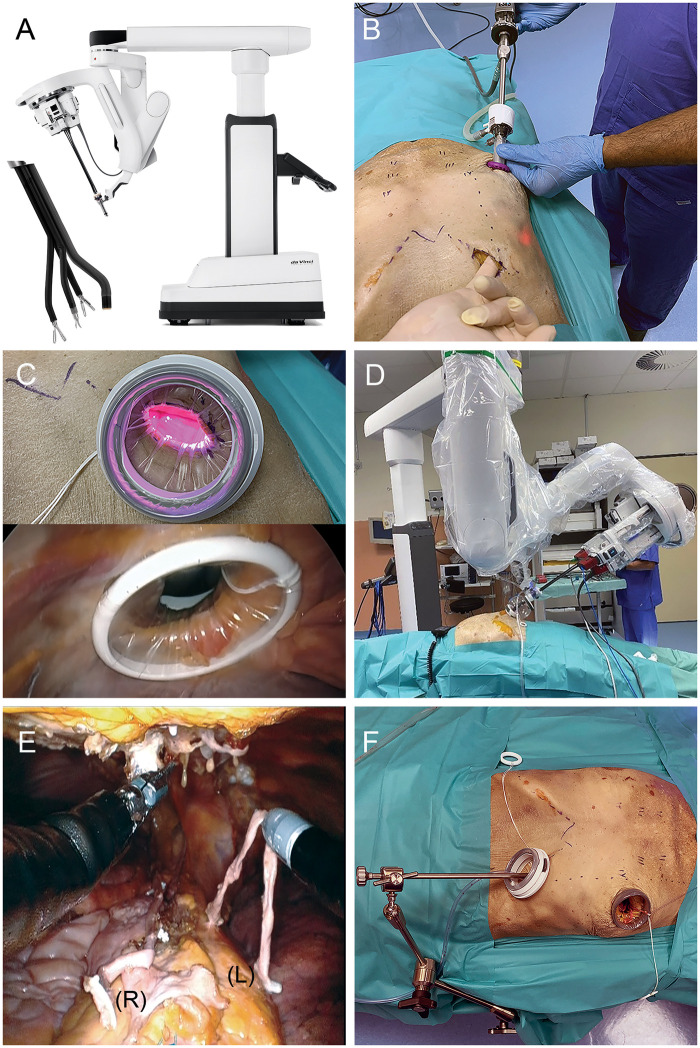
(**A**) da Vinci SP with instruments and camera deployed. (**B**) Skin incision at left subcostal margin (cadaver 2). (**C**) Internal (top) and external (bottom) view of the subcostal access. (**D**) SP system docked. (**E**) Harvested right (R) and left (L) ITA (female cadaver). (**F**) Simulated set-up of a MIDCAB procedure. ITA: internal thoracic artery; MIDCAB: minimally invasive direct coronary artery bypass; SP: Single Port.

The da Vinci SP system provides 3 instruments (Ø 6 mm) and a stereoscopic camera through a single 28-mm port. Instrument elbow joints enable triangulation and independent planar movements with an articulation similar to multiport da Vinci X & Xi systems (Fig. 1A). SP surgeon console user experience is analogous to other da Vinci platforms.

The procedures were performed in 2 human cadavers [male (82 years, body mass index 23), female (74 years, body mass index 24)] and 2 female Yorkshire porcine (43 and 48 kg) in a certified laboratory (DIN EN ISO 9001:2015). The cadaver study was carried out in accordance with ethical standards and the declaration of Helsinki 1964 and its subsequent updates; Institutional Review Board approval was not required (no living human subjects). Porcine procedures were performed under animal research protocol # 2347-23-2018-Ä2 (German State of Brandenburg). The procedure in the male cadaver was performed by robotically trained surgeons (Volkmar Falk, John Michael Smith) and the remaining surgeries by novice robotic surgeons (Joseph Zacharias, Jörg Kempfert).

In the porcine models, we assessed space constraints, the impact of lung ventilation, cardiac motion, vessel pulsations and effective hemostasis.

All models were placed in supine position with the left chest on the OR table edge. The tabletop was ‘flexed’ at the xiphoid process, and a saline bag placed behind the thorax to increase subcostal exposure. A 12-mm observation port was created in the fifth intercostal space (ICS) periareolar, and CO_2_ insufflated at 8 mmHg for inferior diaphragmatic displacement. An endoscope was inserted visualizing the subcostal thoracic entry. A 3 to 4 cm skin incision was created below the subcostal margin between mid-clavicular line and xiphoid process (Fig. 1B). Subcutaneous tissues and oblique muscles were incised until the transverse abdominis fascia was visible. The pleura was entered by tunnelling bluntly with fingers and Metzenbaum scissors below the costal cartilages above the diaphragm under endoscopic view. A small size da Vinci SP Access Port Kit (Intuitive Surgical Inc., Sunnyvale, CA, USA) was placed in the opening (Fig. 1C) and the SP patient-side cart docked (Fig. 1D). The SP camera was configured for an upwards view into the mediastinum. Maryland bipolar forceps, monopolar cautery spatula and scissors, Cadiere forceps and Medium-Large Clip Applier were used. First, the retrosternal tissue was mobilized and this in combination with the CO_2_-pressure provided enough room to safely enter the right pleural space. The SP instrument arm could be clutched freely within the access port which supplied additional reach to harvest the full length of ITA up to the subclavian vein and distally to the bifurcation. Mobilization of the right ITA started at a visible segment. ITA side branches were coagulated with bipolar cautery or occluded with Hem-o-lok clips (Teleflex, Morrisville, NC, USA). The distal end of the RITA was clipped and transected with scissors. The left ITA harvesting was performed in the same fashion. Following, the pericardium was opened, and appropriate coronary target vessels were identified. In the cadaver models, the fifth ICS incision was extended to a small 3 to 4 cm thoracotomy and an XS Alexis wound retractor (Applied Medical, Rancho Santa Margarita, USA) was placed to simulate a MIDCAB procedure (Fig. 1F). Both harvested ITAs were presented through the thoracotomy confirming sufficient length for grafting. Video 1 shows the harvesting procedure in the female cadaver. BITA takedown was performed likewise in both porcine models. Porcine model 2 developed ventricular fibrillation and died of cardiac arrest at 3 min into the mediastinal dissection. The procedure was continued on the deceased animal.

## RESULTS

In all models, BITA mobilization down to the bifurcation was possible. Reaching coronary target vessels (left anterior descending, obtuse marginal) was confirmed through the thoracotomy in the cadavers. Figure 1E shows both ITAs harvested with sufficient length for grafting in cadaver 2. Table 1 summarizes procedure times for all models. Creating the subcostal access and docking the SP system took between 14 and 21 min. All docking in our study was performed by HS and MI which took on average <90 s. Dissection of the mediastinum was longer in the cadaver models due to thymus and fatty tissue adhesions as compared to the porcine model. Total BITA harvest time ranged from 65 to 125 min. In the porcine model, no major bleeding was observed, and hemostasis managed with the available instruments. Nevertheless, one of the porcine models was pulseless during the harvesting procedure.

**Table 1:** Intraoperative times

Model	First skin incision—SP docked (min)	Mediastinal dissection (min)	RITA harvest (min)	LITA harvest (min)	BITA total harvest (min)
Cadaver male	14	30	43	38	81
Cadaver female	16	22	79	42	117
Porcine 1	21	6	56	69	125
Porcine 2	14	5	38	27	65

BITA: bilateral internal thoracic artery; LITA: left internal thoracic artery; RITA: right internal thoracic artery; SP: Single Port.

Some misalignments between the system remote center and the subcostal incision occurred as the instrument arm pivots around a fixed point outside of the body wall while using the SP access port. Minor adjustments of the SP instrument arm were required to resolve this issue.

## DISCUSSION

Our study demonstrates the feasibility of mobilizing BITAs with the da Vinci SP system through a single subcostal incision in cadaveric and live porcine models. The subcostal access and wristed manipulation of the camera tip enabled excellent exposure and visualization of the BITA’s variable course under the curved sternum and facilitated harvesting down to the bifurcations. Despite the limited space in the porcine models with ventilated lungs and cardiac motion it was possible to dissect both ITAs reliably. Conduit lengths were deemed sufficient for multivessel grafting, although the blood-less, unfilled hearts in the cadaver models need to be considered here. We demonstrated a T-graft anastomosis in a previous study [6], which would make use of the mobilized ITA's for multiple graft configurations. Access to the RITA origin at the right subclavian artery was considerably simplified from the subcostal access point. Dissection of the LITA was more challenging towards the distal end since the surgical view was parallel to the vessel making it harder to manage side branches. Reaching the distal aspect of the ITAs at the cardiophrenic angle close to the subcostal incision was eased by the SP access port, since unfolding the instruments for full articulation was possible. This was a limitation in our previous study without the access port [6] and prevented a subcostal approach to the BITA. The required adjustments of the SP system remote centre at this step of the procedure stemmed from a misalignment when working close to the body incision. A custom remote center software feature under development will allow the surgeon to set the remote center at a custom location and enable the SP system to pivot around this software-controlled custom remote center. Interferences and manual user adjustments will be substantially reduced with this.

Some of the issues leading to infrequent use of BITAs in a minimally invasive approach could be addressed with the proposed technique. The SP system made the BITA harvest technically less demanding for all surgeons as reported by them. Even 2 previously robotically untrained surgeons were able to reliably harvest both ITAs in the models after just a short introduction of the technology. Another drawback commonly associated with BITA harvest is the excess time needed for harvesting. Total BITA harvest time ranged from 65 to 125 min in our study. Robotic BITA harvest using the da Vinci Xi by experienced users takes typically <45 min and short learning curves for LITA harvesting have been reported. Hemli *et al.* [9] showed a decrease in LITA harvest times from 39 min in the first 10 patients down to 30 min in the last 10 of 77 patients, while Oehlinger *et al.* [10], had a reduction from 140 to 34 min in a 100-patient cohort. We extrapolate based on this data that similar times should be achievable with the SP system in a subcostal approach.

The subcostal approach might have multiple advantages. First, localizing the coronary target vessels is possible before creating the thoracotomy reducing the risk of a suboptimal access to the coronary targets. Second, a heart stabilizer can be introduced through the subcostal incision for coronary anastomoses to complete a MIDCAB procedure. Third, no additional incisions or traumatic rib-spreading are required avoiding potential damage to the intercostal nerves. Finally, post-operative drains could be placed through the subcostal access averting additional incisions. Limitations of our study result from the chosen models which do not allow for an evaluation of postoperative pain or other relevant outcomes.

For safe adoption of this technique an endoscopic camera should be placed in the fourth or fifth ICS to observe the subcostal entry for safety and efficiency. This might not be required after the surgical team has gained adequate experience, but one could utilize the extra port for laparoscopic instrumentation to assist during ITA takedown (Clip Appliers, Advanced Energy Devices, etc.) as not all robotic instruments available on the Xi platform exist on SP.

## CONCLUSIONS

This small-scale experimental study demonstrates the feasibility of a BITA harvest with the da Vinci SP system through a single left-sided subcostal incision in a cadaveric and porcine model. Some of the issues leading to infrequent use of BITAs in a minimally invasive fashion could be addressed. Robotic harvesting was reported as technically less demanding by the surgeons when compared to direct vision harvesting through a mini-thoracotomy and required no additional rib-spreading. The conduits were sufficient in length for multivessel grafting. Further studies will be required to assess if a subcostal BITA harvest with the da Vinci SP system yields true clinical benefits in humans when combined with a multivessel MIDCAB set-up.

## Data Availability

All relevant data are within the manuscript. Interdisciplinary CardioVascular and Thoracic Surgery thanks Martin Andreas and the other anonymous reviewers for their contribution to the peer review process of this article.
